# Prevalence and Clinical Significance of HIV Drug Resistance Mutations by Ultra-Deep Sequencing in Antiretroviral-Naïve Subjects in the CASTLE Study

**DOI:** 10.1371/journal.pone.0010952

**Published:** 2010-06-03

**Authors:** Max Lataillade, Jennifer Chiarella, Rong Yang, Steven Schnittman, Victoria Wirtz, Jonathan Uy, Daniel Seekins, Mark Krystal, Marco Mancini, Donnie McGrath, Birgitte Simen, Michael Egholm, Michael Kozal

**Affiliations:** 1 Global Development and Medical Affairs, Bristol-Myers Squibb, Wallingford, Connecticut, United States of America; 2 Yale University School of Medicine and Veterans Affairs Connecticut Healthcare System, New Haven, Connecticut, United States of America; 3 454 Life Sciences, a Roche company, Branford, Connecticut, United States of America; University of California San Francisco, United States of America

## Abstract

**Background:**

CASTLE compared the efficacy of atazanavir/ritonavir with lopinavir/ritonavir, each in combination with tenofovir-emtricitabine in ARV-naïve subjects from 5 continents.

**Objectives:**

Determine the baseline rate and clinical significance of TDR mutations using ultra-deep sequencing (UDS) in ARV-naïve subjects in CASTLE.

**Methods:**

A case control study was performed on baseline samples for all 53 subjects with virologic failures (VF) at Week 48 and 95 subjects with virologic successes (VS) randomly selected and matched by CD4 count and viral load. UDS was performed using 454 Life Sciences/Roche technology.

**Results:**

Of 148 samples, 141 had successful UDS (86 subtype B, 55 non-B subtypes). Overall, 30.5% of subjects had a TDR mutation at baseline; 15.6% only had TDR(s) at <20% of the viral population. There was no difference in the rate of TDRs by B (30.2%) or non-B subtypes (30.9%). VF (51) and VS (90) had similar rates of any TDRs (25.5% vs. 33.3%), NNRTI TDRs (11.1% vs.11.8%) and NRTI TDRs (24.4% vs. 25.5%). Of 9 (6.4%) subjects with M184V/I (7 at <20% levels), 6 experienced VF. 16 (11.3%) subjects had multiple TAMs, and 7 experienced VF. 3 (2.1%) subjects had both multiple TAMs+M184V, and all experienced VF. Of 14 (9.9%) subjects with PI TDRs (11 at <20% levels): only 1 experienced virologic failure. The majority of PI TDRs were found in isolation (e.g. 46I) at <20% levels, and had low resistance algorithm scores.

**Conclusion:**

Among a representative sample of ARV-naïve subjects in CASTLE, TDR mutations were common (30.5%); B and non-B subtypes had similar rates of TDRs. Subjects with multiple PI TDRs were infrequent. Overall, TDRs did not affect virologic response for subjects on a boosted PI by week 48; however, a small subset of subjects with extensive NRTI backbone TDR patterns experienced virologic failure.

## Introduction

Low abundance drug resistant HIV variants at levels as low as 1% of the circulating viral quasispecies can be detected in antiretroviral (ARV)-naïve individuals by sensitive and quantitative genotyping technologies [1]–[6]. These low abundance drug resistant variants have been shown to potentially impact clinical responses in individuals initiating non-nucleoside reverse transcriptase based ARV therapy [7]–[14]. However, other studies did not find a strong association with clinical responses [15]–[16]. The conflicting results may have been the result of both the different sensitive genotyping methods used in the investigations and the disparate study populations that received heterogeneous antiretroviral regimens. Few studies have been able to control for the antiretroviral regimens used when investigating the impact of low abundance resistant variants on clinical outcomes.

Although low abundance resistant variants have been shown to be important in some HIV-infected populations, there are only limited data available on the rate of low abundance transmitted drug resistance (TDRs) mutations in diverse ARV-naïve populations infected with B and non-B HIV subtypes [17]. There are also little data on the clinical significance of low abundance variants possessing TDRs in subjects who begin therapy with ritonavir-boosted protease inhibitor (PI)-based therapy. An important question in the HIV field currently is how persons who harbor low abundance ARV-resistant variants respond to different ARV treatment regimens.

The CASTLE study was a randomized open label study for treatment naïve persons that compared the efficacy of atazanavir/ritonavir (ATV/r) with lopinavir/ritonavir (LPV/r), each in combination with tenofovir-emtricitabine (TDF/FTC) in ARV-naïve subjects from 5 continents [18]. The previously reported results demonstrated that ATV/r was non-inferior to LPV/r at Weeks 48 and 96 [18]–[19]. Both regimens achieved consistent response rates regardless of HIV subtype or number of baseline protease gene polymorphisms present [18]–[19]. In addition, both regimens had similar low rates of selection of new ARV resistance mutations [18]–[21].

In this study, we report the baseline rate of low abundance drug resistant HIV variants detected by ultra-deep sequencing in persons infected with B and non-B subtypes from 5 continents (Africa, Asia, Europe, North and South America). The association of baseline drug resistant variants detected by ultra-deep sequencing on virologic responses at Week 48 in the CASTLE study is explored.

## Results

### Baseline Characteristics and Rate of TDRs

A total of 148 samples were recovered from the CASTLE study sample archives for UDS. All 53 VF specimens and 95 VS specimens were sequenced. The baseline characteristics of these 148 did not differ from the parent study population: mean age 35 years, 34% female, median CD4 cell count 191 cells/mm^3^. Baseline viral loads ranged from 3030 to >750,000 copies/mL with a median viral load of 132,500 copies/mL.

**Figure 1:**
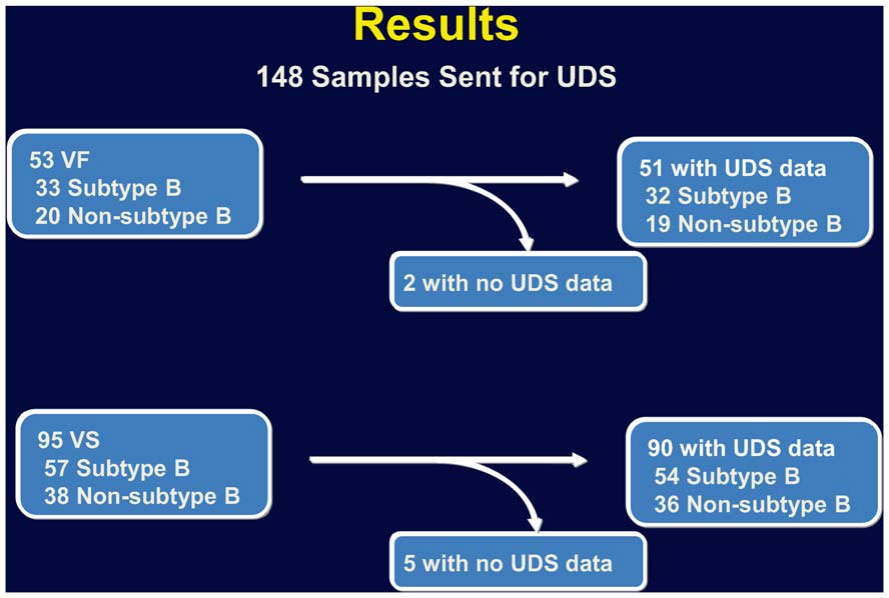
Results of Ultra Deep Sequencing Analysis. 148 samples recovered and sent for UDS: Subtype B (n = 90), Non-B (n = 58); 53 were VF and 95 were VS. 141 samples had UDS data: Subtype B (n = 86), Non-B (n = 55) 51 VF and 90 VS. Of the 7 samples without UDS data, 2 had partial UDS data and were not included; 3 samples were exhausted; and 2 had low viral loads and unable to amplify. VF: Virologic Failure, VS: Virologic Success.

Of the 148 baseline samples, 90 were subtype B, and 58 were non-B subtype (A, AE, C, BF and F1). UDS results were obtained on 141 of the samples (51 VF and 90 VS, 86 B and 55 non-B subtypes) (Figure 1). Seven samples could not undergo UDS but were genotyped by standard genotyping; 3 were non-B (AE) subtype, and 4 were B subtype. One sample had UDS but no standard genotype. Samples without UDS data had 1) only partial UDS data obtained, 2) low viral loads, and/or 3) had undergone prior freeze-thaw cycles for other analyses (Figure 1).

Overall, for subjects with UDS results, 30.5% had a TDR mutation at baseline detected by UDS, and 15.6% had only TDR(s) at <20% rate in the viral population (Table 1). There was no difference in the rate of TDRs by B (30.2%, 26/86) or non-B subtypes (30.9%,17/55). At least one TDR was identified in a sample from subtypes B, AE, C, and BF. There were only one subtype A and one subtype F1 sample, neither with a TDR. Samples from all 5 continents had TDRs by UDS: Africa 8/28 (28.6%), Asia 2/8 (25%), Europe 2/11 (18.2%), North America 8/19 (42.1%), and South America 23/75 (30.7%). The overall rate of any TDR by standard genotyping was 18/147 (12.2%) (Table 2). There is no difference between VFs (6/52) and VSs (12/95) regarding the rate of TDRs by standard sequencing, P = NS. UDS detected more TDRs overall and by specific ARV class than standard genotyping.

**Table 1:** Prevalence of low and high abundance Transmitted Drug Resistance Mutations (TDRs) by antiretroviral drug class.

WHO TDR Mutation Class	<20% of viral Population^1^	≥20% of viral Population^2^	Subjects with any TDR
Any TDR	22 (15.6%)	21 (14.9%)	43 (30.5%)
N(**t**)RTI	19 (13.5%)	16 (11.3%)	35 (24.8%)
NNRTI	12 (8.5%)	4 (2.8%)	16 (11.3%)
PI	11 (7.8%)	3 (2.1%)	14 (9.9%)

N(**t**)RTI(s)- nucleoside(tide) reverse transcriptase inhibitor; NNRTI – non-nucleoside reverse transcriptase inhibitor, PI – protease inhibitor. Samples with multiple TDRs present with at least one TDR representing ≥20% of the viral populations were scored as having ≥20% of the viral population (e.g. M184V at 5% and a T215Y at >20% are in the ≥20% column). Only samples where all TDRs were at <20% levels are represented in the <20% column; 21 subjects had at least 1 TDR at high level ≥20%: 6 VFs and 15 VSs. There was no difference between VFs (6/51) and VSs (15/90) regarding the rate of high level (≥20%) TDRs. P-value = 0.47 Fisher's exact test.

1 Only TDRs occurring at <20% of the viral population.

2 At least 1 TDR occurring at ≥20% of the viral population.

**Table 2:** Prevalence of transmitted drug resistance (TDR) mutations by Ultra-deep Sequencing (UDS) and by standard genotyping (SG).

WHO TDR ARV class	% of Subjects with a TDR by UDS (n = 141)	% of Subjects with a TDR by SG (n = 147)
Any TDR	30.5%	12.2%
N(**t**)RTI	24.8%	9.5%
NNRTI	11.3%	2.0%
PI	9.9%	2.0%

N(**t**)RTI – nucleoside(tide) reverse transcriptase inhibitor; NNRTI – non-nucleoside reverse transcriptase inhibitor, PI – protease inhibitor. 141 subjects with UDS data; 147 subjects with standard genotypic data.

### Specific TDR Antiretroviral (ARV) Mutation Class

Of the 141 subjects with a UDS result, 35 (24.8%) had a nucleoside(tide) reverse transcriptase inhibitor N(t)RTI(s) TDR; 26 of these had a thymidine analog mutation (TAM) [22], 9 had a M184V/I, and 2 had a variant with a K65R. Sixteen subjects (11.3%) had non-nucleoside reverse transcriptase inhibitor (NNRTI) TDR; of these, 15 had one or more of K103N, Y181C/I or G190A/E. Fourteen subjects (9.9%) had a protease inhibitor (PI) TDR. Eighteen subjects (12.8%) had TDRs from at least two different ARV classes.

Of the 14 subjects with a PI TDRs, 11 (78.6%) had PI TDRs at a <20% levels. The majority of PI TDRs (10/14) were present as solitary PI TDRs (e.g. 24I, 32I, 46I or 58E). Only 4 subjects had >1 PI TDRs: 3 clade B samples with 85V+90M; 46I+84V; and 46I+ 58E+84V+85V+90M; and one clade C sample with 32I+46I. The PI TDR patterns were interpreted for all subjects with the Stanford HIVdb algorithm [23]. Only one subject had high level resistance to the PI used (ATV), possessing TDRs 46I, 58E, 84V, 85V, and 90M.

### Association of TDRs with Virologic Success (VS) and Virologic Failure (VF)

**Figure 2:**
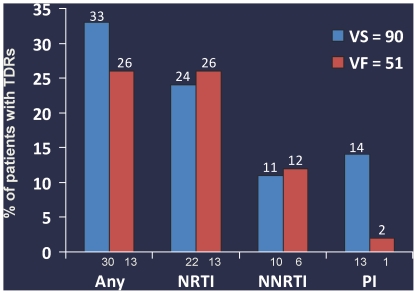
Transmitted Drug Resistance by ARV Class and Virologic Outcome. Transmitted Drug Resistance by ARV Class and Virologic Outcome: Note: TDRs are not mutually exclusive (i.e. a subject could have a TDR from more than one ARV class). VS: Virologic Success, VF: Virologic Failure N(t)RTI(s)- nucleoside(tide) reverse transcriptase inhibitor; NNRTI – non-nucleoside reverse transcriptase inhibitor, PI – protease inhibitor. Any: Any transmitted drug resistance mutations. Any TDR: p-value = 0.35, NRTI: p-value = 1, NNRTI: p-value = 1, PI: p-value = 0.02.

Among the 141 samples with a UDS result, there was no difference in the rates of TDRs among those with VS 33.3% (30/90) and VF 25.5% (13/51) by Week 48 (P = 0.35). Rates of TDRs by ARV class in those with VF and VS are shown in Figure 2. Although the VS group had more PI TDRs by UDS than the VF subjects (14.4% vs. 2.0%, P = 0.02) most PI TDRs were found in isolation, had low Stanford HIVdb scores [23] (e.g. 24I, 32I, 46I, and 58E), and were at <20% of the viral population. One patient had multiple PI and N(t)RTI TDRs in high abundance (>20%) and experienced virologic failure.

### Specific TDRs known to affect the N(t)RTI backbone

Considering specific TDRs that could impact virologic response to the N(t)RTI backbone, TDF/FTC, used in the study, 9 (6.4%) of the subjects had M184V/I identified by UDS at baseline. Seven of 9 M184V/I were at <20% levels and were not detected by standard genotyping. Six of the 9 experienced VF; 4 of these also had TAMs, and 1 had K65R+TAMs (Table 3). Specific TDR patterns and levels for subjects with a M184V/I and K65R are shown in Table 4. Sixteen subjects (11.3%) had multiple TAMs by UDS and 7 experienced VF. Three (2.1%) subjects had M184V and multiple TAMs, and all were VF; 2 subjects had a K65R at baseline by UDS (1 VF and 1 VS) (Table 3).

**Table 3:** Virologic outcome at Week 48 and specific TDR at Baseline known to affect NRTI Backbone.

Specific TDR Mutations	VS (n = 90)	VF (n = 51)	Total # of Subjects with Specific TDR (n = 141)
M184V/I +/− TAMs +/− K65R	3	6	9
>1 TAMs	9	7	16
K65R	1	1	2

Thymidine Analog Mutations (TAMs); Virologic Success at week 48 (VS), Virologic Failure at week 48 (VF). Transmitted Drug Resistance Mutations (TDRs); 9 subjects with M184V/I +/− TAMs and/or +/− K65R at baseline. 16 subjects with >1 TAMs at baseline. 2 subjects with K65R at baseline.

**Table 4:** Specific TDR patterns and levels for subjects with a M184V/I and/or K65R +/− TAMs.

ID	Clade	48 w	VL	TAMs	TDRs at baseline with UDS
1 - ATV	B	VF	334,000	1	**RT: M184V(1%)**, **T215F(1%)**, G190A(0.9%)**PR:** I13V(1.5%), L63P(99.1%), A71T(96.9%)
2 - ATV	B	VS	545,000		**RT:** D67G(1.3%), **M184V(0.9%)**, K103N(67.6%)**PR:** L10F(1.6%), L10I(95.4%), I13V(1.3%), K20V(1.3%),**V32I(1.4%)**, M36I(3.6%), L63P(0.9%), V82I(98.6%), I93L(99.2%)
3 - ATV	B	VF	193,000	5	**RT: M41L(1.72%)**, **D67N(1.87%)**, **K70R(1.67%)**, **M184V(5.9%)**, **T215Y(20.49%)**, **K219Q(26.99%)** **PR:** I13V(23.23%), K20R(1.42%), M36I(98.11%), I62V(99.18%), L63P(100%), A71T(29.74%), I93L(96.64%)
4 - ATV	B	VS	168,000		**RT: M184I(1.25%)**, V179D(26.85%)**PR:** M36I(1.16%), **M46I(1.08%)**, L63P(100%), V77I(6.49%), **I84V(1.11%)**
5 - ATV	B	VF	158,000	2	**RT: M41L(94.79%)**, **M184V(96.76%)**, **T215Y(97.69%)** **PR:** L10F(99.21%), **M46I(20.87%)**, Q58E(91.85%), I62V(8.48%), L63P(98.8%), I64V(2.26%), V77I(58.2%), **I84V(36.63%)**, I85V(45.4%), **L90M(46.63%)**, I93L(91.49%)
6-LPV^#^	C	VF	750,000	3	**RT: K65R(3.11%)**, **M184V(13.79%)**, **L210W(9.27%)**, **T215Y(9.1%)**, **K219Q(4.66%)**, G190A (4.57%)**PR:** L10I(3.22%), L10I(77.13%), I13V(72.43%), M36I(82.5%), L63P(1.06%), H69K(94.02%), I93L(45.59%)
7 - LPV	B	VF	35,000		**RT: M184I(3.37%)** **PR:** I62V(98.93%), L63P(100%), I64L(100%), V82I(90.91%), I93L(94.44%)
8 -LPV	B	VF	37,000		**RT: M184V(95.98%)**, K103N(14.24%)**PR:** I64V3(99.82%)
9 - LPV	C	VS	38,100	1	**RT:** V75A(1.95%), **M184V (1.43%)**, **L210W(1.3%)**, K103R(2.98%)**PR:** K20M(98.93%), M36I (97.89%), K43T(1.31%), I62V(100%) L63P(100%), H69K(100%), I93L(99.76%)
10 - ATV	BF	VS	8840	6	**NRTI: M41L(7.47%)**, **K65R(7.85%)**, **D67N(33.78%)**, **K70R(6.34%)**, Y118I(1.65%), **L210W(16.74%)**, **T215C(20.98%)**, **K219N(22.46%)** **NNRTI:** K103N(16.36%), K103R(10.27%), V179D(73.28%), Y181C(2.34%)**PI:** L10R(2.51%), M36I(91.67%), **M46I(3.3%)**, D60E(2.51%), L63P(3.28%), I64L(2.88%), V77I(1.79%), V82I(92.67%), I93L(90.53%)

VF: Virologic Failure VS: Virologic Success NRTI: Nucleoside Reverse Transcriptase Inhibitor NNRTI: Non-Nucleoside Reverse Transcriptase Inhibitor PI: Protease Inhibitor. TAMs: Thymidine Analogue Mutations. HIV VL: HIV Viral Load. TDRs: Transmitted Drug Resistance Mutations. 6-LPV^#^ has both a M184V and a K65R TDR. **Bold: M184V, TAMs, Major PI mutations according to HIV Stanford Database.**

## Discussion

Among a representative sample of ARV-naïve subjects from the CASTLE study, HIV variants possessing TDR mutations were commonly detected by UDS. Similar rates of TDRs were identified in B and non-B subtypes. Although the study was not designed to evaluate TDRs by continent nor specific subtype, at least one sample from ARV-naïve subjects from 5 continents (Africa, Asia, Europe, North and South America) had a TDR by UDS. These data suggest that among subjects entering large international clinical trials, TDRs are common in ARV-naive persons from diverse populations and infected with B and non B subtypes. Many of these subjects harboring HIV variants with TDRs were not detected by standard genotyping. The overall TDR rate of 30.5% by UDS among ARV-naïve subjects in our study is similar to other investigations that reported rates between 28 to 33% depending on the populations studied and the sensitive genotyping methods used to identify low abundance resistant variants [14]–[16], [24].

When considering TDRs both overall and by specific ARV class, TDRs were not associated with a lower virologic response for subjects initiating a boosted PI-based regimen. However, a small subset of subjects with specific N(t)RTI TDR patterns that could impact the activity of the TDF/FTC backbone used in the study did experience a higher rate of virologic failure. Interestingly, subjects with a PI TDRs identified by UDS at baseline did well on a boosted PI regimen. Possible reasons for this apparently non-intuitive finding include: 1) most PI TDRs were found in isolation (few subjects had multiple PI TDRs), 2) the PI TDRs identified had low Stanford HIVdb scores [23] (e.g. 24I, 32I, 46I, and 58E and some of these have been previously described to be naturally occurring polymorphisms within the HIV-1 protease gene [2], and 3) the majority of PI TDRs were at very low levels of the viral population (<20% of the population). Thus, PI TDRs identified by UDS may have little impact on virologic response when the TDR is found in isolation and at low levels, especially in a person initiating a boosted PI regimen with a high genetic barrier to resistance. The PI TDR patterns were interpreted for all subjects with the Stanford HIVdb algorithm [23]. Only one subject had high-level resistance to the PI used (ATV). This subject experienced VF and had multiple PI TDRs (46I, 58E, 84V, 85V, 90M) in high abundance (>20% levels). This subject demonstrates the previously known information that multiple PI TDRs present at high levels can impact virologic responses to boosted PI based regimens [18]–[21], [25]–[29].

There was no difference in the rate of NNRTI TDRs in subjects with VS or VF with approximately 11% having a TDR NNRTI mutation. This data suggests that if a person harbors NNRTI resistant variants, a boosted PI regimen may be successful at suppressing the resistant variants. It has been previously shown that many of the low abundance resistant variants identified by UDS may not affect virologic outcomes if the resistant variants identified are only resistant to ARVs that are not part of the antiretroviral regimen being employed [14], [30].

Although there was no difference in the rate of N(t)RTI TDRs in those subjects with VS or VF, there were subjects with extensive N(t)RTI TDR mutations which are known to impact responses to TDF/FTC. There was a high rate of VF in the small number of subjects with a M184V, even when the TDR was present at <20% levels of the quasispecies and missed by standard genotyping methods. Subjects with multiple TAMs also had disproportionate rates of VF. These results suggest that M184V and specific patterns of N(t)RTI mutation patterns may contribute to predicting response to a boosted PI regimen. However, the number of subjects in this study is small, and more studies should be done to investigate the impact of specific N(t)RTI TDR patterns present in low abundance on virologic responses in subjects initiating boosted PI regimens.

A strength of this study was that the patient population was recently sampled (enrolled between 2005–2006), well characterized, and represented multiple diverse ARV-naïve groups and HIV subtypes. A limitation of this study was that it was a retrospective case control study with the inherent limitations of such a design. The study design allowed us to explore the association of TDRs with clinical responses with a boosted PI regimen and the rate of TDRs in B and non B subtypes. However, investigations into the prevalence of TDRs across continents or patterns of specific TDRs by HIV subtype could not be definitely defined given that the numbers in each category were too few. Future studies should be performed to determine the prevalence of TDRs by UDS in larger retrospective and prospective studies.

Also, recent reports have suggested that the absolute number of viral variants with a TDR mutation, or “mutational load,” may impact the time to virologic failure in a person on therapy [31]–[33]. Although our study was not designed to specifically determine mutational load association with virologic response, subjects with high mutational loads were generally more likely to experience VF (data not shown). Thus, several factors should be considered when evaluating minor variants effect on treatment response, such as, the specific TDR resistance conferring effect, the proportion of variants possessing the mutation (mutational load), the total number of TDRs present that affect the regimen and whether these TDRs are linked on same viral genome, and the genetic barrier of the specific ARV regimen being used. Virologic outcome based on baseline minority variants is likely multifactorial, and is not simply based on presence or absence of a mutation or a simple mutation percentage, as some mutations and mutational loads likely can be suppressed by specific regimens, in this case a boosted PI regimen [31]–[33].

Further studies should be performed to determine how the viral factors of mutational load, mutation linkage (mutations within the same viral genome), and specific mutational patterns interact and impact treatment responses to the many different antiretroviral regimens now used in the clinic. These important virologic parameters will need to be better defined, and the resistance interpretation algorithms accordingly adapted before sensitive genotyping technologies can be incorporated into routine HIV clinical care.

In summary, among a representative sample of ARV-naïve subjects in the CASTLE study, transmitted drug resistance mutations identified by ultra-deep sequencing were common, and B and non-B HIV-1 subtypes had a similar rate of TDRs. TDRs identified by UDS did not affect virologic response for subjects on a boosted PI. However, a small subset of subjects with extensive N(t)RTI backbone TDR patterns were likely to experience virologic failure. Further investigations should be performed to determine the prevalence of TDRs in diverse populations of HIV infected persons across the different regions of the world. Studies should also be done to determine which ARV regimens are best at suppressing HIV variants possessing TDRs at different variant levels and mutation patterns.

## Methods

### Trial population and sample selection

The CASTLE study was a 96 week study comparing the antiviral efficacy and safety of ATV/r with LPV/r, each in combination with TDF/FTC in HIV-infected treatment-naïve subjects. The primary objective of the study was to compare the proportion of subjects with HIV RNA levels <50 c/mL at Week 48 between the ATV/r+TDF/FTC and LPV/r+TDF/FTC regimens. The primary analysis of the study confirmed the hypothesis that ATV/r/TDF/FTC was non inferior to LPV/r/TDF/FTC with 78% and 76% of subjects respectively, having HIV RNA <50 c/mL at Week 48. Subjects were enrolled from 5 continents (Africa, Asia, Europe, North and South America) representing 28 countries and had given informed consent [18]–[19].

Based on prior studies [14], [24], [34], our research hypothesis was that low frequency drug resistant HIV variants would be detected in baseline samples in 10–20% of subjects with virologic failure. A retrospective case control study was performed to determine the baseline rate of low (<20% of the viral population) and high abundance (≥20%) drug resistant HIV variants by ultra-deep sequencing in subjects with virologic failure (VF) and virologic success (VS) defined by Confirmed Virologic Response (CVR) for HIV RNA <400 copies/mL by Week 48 [18]–[21]. Baseline specimens were selected from all subjects with VF by week 48 (N = 53: 27 from ATV/r arm and 26 from LPV/r) and from 95 VS subjects (n = 50 from ATV/r arm and n = 45 from LPV/r arm). VS samples were randomly selected using stratified sampling by baseline HIV RNA and CD4 so that the VS had similar baseline HIV RNA and CD4 as the VF subjects.

Subjects were selected from all 5 continents, and 6 different HIV subtypes were represented. Baseline demography, CD4 cell count and viral load values, and baseline HIV genotype by standard genotyping (Monogram Biosciences, CA) were obtained from the clinical trial database.

### Ultra-deep sequencing (UDS)

Coded baseline plasma samples were sent to Yale for ultra-deep sequencing (UDS) using 454 Life Sciences sequencing center (Branford, CT). The method for UDS has been described elsewhere [14], [34], [35]. However, in brief, three gene-specific overlapping cDNA per sample were generated from extracted RNA and subjected to 40 cycles of PCR. These products were then used to make eight partly overlapping amplicons covering the HIV-1 *pol* gene (protease and reverse transcriptase gene codons 1–236). The amplicons were purified with AMPure magnetic beads (Agencourt, Beverly, MA) and quantitated by PicoGreen fluorescence (Invitrogen). After equimolar pooling of amplicons, clonal amplification on beads was performed using UDS sequencing kits (Roche/454 Life Sciences, Branford, CT). Sample emulsions were pooled, enriched, and counted on a Multisizer3 Coulter counter. Approximately 30,000 beads per sample were prepared for UDS. Sequencing was performed on a Genome Sequencer FLX (Roche/454 Life Sciences). An average of 1700 reads per nucleotide position was obtained for this set of samples, which allowed for accurate detection of variants down to approximately 1% when viral load is >10,000 copies/mL [14], [34]. In this study 5.4% (8/148) of samples had viral loads <10,000; 5 had successful UDS (viral loads of 9680, 7030, 8840, 5870, and 9190 copies/mL) with good sequencing depth coverage (ave. ∼1500 reads) and were included. Given stochastic effects of RNA sampling for samples with VL<10,000 copies/mL, the proportion of variants in these samples reflect the proportion of mutations within the PCR amplicons sequenced and may or may not reflect the proportion in plasma [14], [34]. Amplicon Variant Analyzer software was used to align all amplicon sequence reads to a consensus sequence. The limit of detection for TDRs by UDS was restricted to ≥1% of the viral population [14], [34], [35].

### Standard genotypic resistance profiles

Standard genotypic resistance mutation results were determined using Monogram Biosciences GeneSeq technology (South San Francisco CA) [18]–[21].

### Transmitted Drug Resistance Definition

The 2009 World Health Organization reference list of transmitted drug resistance mutations (TDRs) was used to evaluate for the rate of mutations detected by standard and ultra-deep sequencing [17].

### Statistical Methods

Proportions of subjects with TDRs detected by UDS were presented overall and by HIV subtypes, regions, and outcomes at Week 48 (VF or VS). Specific TDR mutation classes (NRTI, NNRTI and PI) were presented similarly. The proportions of subjects with TDRs (all mutations and by specific ARV class) at <20% of the viral population were further calculated. Proportions of subjects with TDRs by B vs. non-B subtypes were compared using Fisher's exact test [36]. Proportions of subjects with TDRs (all mutations and by specific ARV class) by VF vs. VS at Week 48 were also compared using Fisher's exact tests.

The parent CASTLE study is registered with ClinicalTrials.gov, number NCT00272779.
